# The Synergistic Effect of Tacrolimus (FK506) or Everolimus and Azoles Against *Scedosporium* and *Lomentospora* Species *In Vivo* and *In Vitro*

**DOI:** 10.3389/fcimb.2022.864912

**Published:** 2022-04-14

**Authors:** Zikuo Wang, Mei Liu, Luyao Liu, Linyun Li, Lihua Tan, Yi Sun

**Affiliations:** ^1^ Health Science Center, Yangtze University, Jingzhou, China; ^2^ Department of Dermatology, Jingzhou Hospital, Yangtze University, Candidate Branch of National Clinical Research Center for Skin and Immune Diseases, Jingzhou, China; ^3^ Clinical Lab, Jingzhou Hospital, Yangtze University, Jingzhou, China

**Keywords:** Immunocompromised adults, Azoles, Tacrolimus, Everolimus, *Scedosprium*

## Abstract

*Scedosporium* and *Lomentospora* infections in humans are generally chronic and stubborn. The use of azoles alone cannot usually inhibit the growth of these fungi. To further explore the combined effect of multiple drugs and potential mechanisms of action, we tested the antifungal effects of tacrolimus (FK506) and everolimus in combination with azoles *in vitro* and *in vivo* on 15 clinical strains of *Scedosporium*/*Lomentospora* species and detected the level of Rhodamine 6G, ROS activity, and apoptosis. The *in vitro* results showed that the combinations of tacrolimus with itraconazole, voriconazole, and posaconazole showed synergistic effects on 9 strains (60%), 10 strains (73%), and 7 strains (47%), respectively, and the combinations of everolimus with itraconazole, voriconazole, and posaconazole showed synergistic effects on 8 strains (53%), 8 strains (53%), and 7 strains (47%), respectively. The synergistic effects might correspond to the elevated ROS activity (the tacrolimus + itraconazole group compared to the itraconazole group, (*P* < 0.05)), early apoptosis (itraconazole (*P* < 0.05) and voriconazole (*P* < 0.05) combined with everolimus), and late apoptosis (the tacrolimus + itraconazole group compared to the itraconazole group, (*P* < 0.01); the tacrolimus + posaconazole group compared to the posaconazole group, (*P* < 0.05)), but not inhibition of efflux pump activity. Our *in vitro* results suggested that a combination of tacrolimus or everolimus and azoles have a synergistic effect against *Scedosporium/Lomentospora*. The synergistic mechanisms of action might be triggering excessive ROS activity and apoptosis*. In vivo*, the survival rate of *G. mellonella* (sixth instar larvae) was significantly improved by tacrolimus alone, everolimus alone, azoles alone, and tacrolimus and everolimus combined with azoles separately (*P* < 0.05 for the tacrolimus group; *P* < 0.01 for the everolimus group and the itraconazole group; *P* = 0.0001 for the tacrolimus and posaconazole group; *P* < 0.0001 for other groups except the everolimus and itraconazole group, everolimus and posaconazole group, and tacrolimus and itraconazole group). From the results, we infer that the combination of tacrolimus or everolimus with azoles has obvious synergistic effect on *Scedosporium/Lomentospora*, and might enhance the level of apoptosis and necrosis. However, the synergistic effects were not related to the efflux pump. In conclusion, from our *in vitro* and *in vivo* study, tacrolimus and everolimus combined with azoles may have a synergistic effect in the treatment against *Scedosporium/Lomentospora*, improving the drug activity of azoles and promoting a better prognosis for patients.

## Highlights

In recent years, the infection rate of *Scedosporium/Lomentospora* in humans has increased. Because these fungi are resistant to several antifungal drugs, it is urgent to find other drugs and use them in combination with antifungal drugs to enhance their therapeutic effect. We initiated the current *in vitro* and *in vivo* study to confirm the potential synergistic effects and possible mechanisms of action of tacrolimus or everolimus combined with azoles against *Scedosporium* and *Lomentospora*. The results, hereby, helped gain insight into the effect and mechanism of action of tacrolimus or everolimus combined with azoles and provided evidence for using these regimes to combat such types of rare infections.

## Introduction

Invasive fungal infections are life-threatening, especially for immunocompromised and hospitalized patients with severe illnesses (Bouchara and Papon, 2019). *Scedosporium* species are aggressive and opportunistic human pathogens, causing a wide spectrum of infections ranging from subcutaneous infections or spreading *via* the lymphatic system to disseminated infections with systemic involvement. These pathogens are known for high rates of therapeutic failures and relapses, and some of them possess a pan-antifungal resistance with mortality rates of up to 80% in immunocompromised patients (Ramirez-Garcia et al., 2018). In addition, the diagnosis of this infection is relatively difficult because its clinical characteristics and histopathology are similar to aspergillosis, fusariosis, and other relatively common hyalohyphomycoses (Tortorano et al., 2014; Manikandan et al., 2019). Therefore, identifying other drugs to be used in combination with antifungal drugs to treat *Scedosporium* and *Lomentospora* infections has guiding significance for clinical practice. Usually, the calcineurin inhibitor tacrolimus is used in immunotherapy after renal transplantation (Schutte-Nutgen et al., 2018). In the previous clinical case reports, we found that the use of immunosuppressant tacrolimus in the treatment of patients with primary infection of *Scedosporium* and the use of voriconazole in the treatment of fungal infection had a positive effect that patients were cured within a year (Strunk et al., 2015; Centellas Perez et al., 2019). The *in vitro* combination of tacrolimus and azoles has shown synergistic effects on a variety of fungi, such as *Aspergillus* species*, Candida* species, and *Cryptococcus* species (Denardi et al., 2015; Lee et al., 2018; Schwarz and Dannaoui, 2020). Everolimus is an mTOR inhibitor. It has been approved to treat several types of malignant tumor, such as breast cancer, pancreatic cancer and lung cancer. (Lui et al., 2016). Everolimus is also approved to treat transplant rejection (Hauser et al., 2021). There is no relevant study about antifungal capacity of everolimus. Considering the relevance of *Scedosporium* and *Lomentospora* species as important pathogens causing hyalohyphomycosis and the potential therapeutic effect of the combination of tacrolimus and everolimus with azoles, we undertook the present study. *G. mellonella* (sixth instar larvae) models have been used to explore the effect of drug combinations on *Scedosporium* and *Lomentospora* (Yang et al., 2022). The model was also used to evaluate the combination effect of tacrolimus and everolimus with azoles *in vivo*.

## Materials and Methods

### Fungal Strains, Antifungals, and Chemical Agents

The *Scedosporium* and *Lomentospora* strains were grown on SDA at 37°C. All strains were clinical isolates. *Scedosporium aurantiacum* (CBS 116910), *Scedosporium boydii* (CBS 101.22), *Scedosporium dehoogii* (CBS 117406), and *Scedosporium apiospermum* (CBS 116899) were kindly provided by Sybren De Hoog, from the Westerdijk Fungal Biodiversity Institute, Utrecht, the Netherlands. *S. boydii* (M013, accession no. OL348451), *S. apiospermum* (SDB-l, accession no. OL348466), *Scedosporium minutisporum* (SDB-I, accession no. OL348464), *Lomentospora prolificans* (D13h, accession no. OL348462), *S. apiospermum* (D13g, accession no. OL348477), *S. apiospermum* (D13f, accession no. OL348480), *S. apiospermum* (D13d, accession no. OL348483), *S. boydii* (D13c, accession no. OL348500), *S. boydii* (D13b, accession no. OL348498), and *S. boydii* (D13a, accession no. OL348503) were kindly provided by Professor Qiaoyun Lu from the Xiangyang Central Hospital. *S. apiospermum* (L12E, accession no. OL348504) was the strain isolated from a hyalohyphomycoses patient who was cured using tacrolimus (1 mg, bid) and voriconazole (200 mg bid for the first day, then 200 mg qd) for six months. *Candida parapsilosis* ATCC 22019 was included to ensure quality control for the *in vitro* antifungal test. Antifungal and chemical agents, including tacrolimus (NO. S5003), everolimus (No. S1120), itraconazole (No. S2476), voriconazole (No. S1442), and posaconazole (NO. S1257) were purchased in the powder form from Selleck Chemicals, Houston, TX, USA and dissolved in dimethyl sulfoxide to prepare stock solutions (6400 µg/ml). Dihydrorhodamine 123 (No. D1054), a fluorescent dye purchased from Sigma-Aldrich in powder form, was dissolved in dimethyl sulfoxide and a stock solution (6400 µg/ml) was prepared. Rhodamine 6G (No. R4127), purchased from Sigma-Aldrich in powder form, was dissolved in dimethyl sulfoxide to prepare a stock solution (2000 µM). Annexin V-FITC/PI apoptosis detection kit (40302es20) was purchased from Yeasen Biotechnology (Shanghai) Co., Ltd. (Yeasen).

### *In Vitro* Combined Drug Sensitivity

The chequerboard method and proportional dilution method were carried out using a broth microdilution method according to the Clinical Laboratory Standards Institute guidelines (CLSI, 2008; Sun et al., 2020). Briefly, the stock solutions of tacrolimus, everolimus, posaconazole, voriconazole, and itraconazole were diluted using the RPMI-1640 liquid medium. The concentration range of tacrolimus and everolimus was 0.5-32 μg/ml and that of the three different azoles was 0.125-16 μg/ml. Azoles were added to tacrolimus and everolimus in 96-well plates, separately. Rows A-G and columns 2-9 were the test samples, and 2 times the final concentration was added to each well. Columns 2-9 were posaconazole, voriconazole, and itraconazole. Twice the final concentration of tacrolimus and everolimus was added to columns A-G to ensure that the correct final concentration. Both positive and negative controls were included. The conidia of the 15 strains were collected and adjusted to a concentration of 3-5 × 10^6^/ml using a hemocytometer and diluted with RPMI-1640 to a final concentration of 3-5 × 10^4^/ml. About 100 μL of the fungal suspension was added and the samples were incubated in a 35°C incubator for 48 hours. The tests were repeated 3 times, the average value was taken, and the results were recorded. The lowest drug concentration corresponding to 100% inhibition of growth was defined as MIC. The combined effects of drugs were categorized based on the fractional inhibitory concentration index (FICI). The formula of FICI used was: FICI = (Ac/Aa) + (Bc/Ba), where Ac and Bc were MICs of azoles combined with tacrolimus and everolimus, respectively, and Aa and Ba were MIC of the antifungal drugs A and B, respectively. FICI was categorized as follows: ≤0.5 for synergy, >0.5 and ≤4 for no interaction, and >4 for antagonism.

### ROS Activity

From the result of antifungal test, all the combination groups showed synergy against *S. apiospermum* (L12E) (**Table 1**), so we chose this strain for the potential synergistic mechanism study. The cultured *S. apiospermum* (L12E) was accurately counted and dissolved in 10 ml Sabouraud dextrose broth (SDB) to ensure a final spore concentration of 5 × 10^6/^ml. Based on the results of the antifungal susceptibility test, solutions of appropriate concentrations of azoles, tacrolimus, and everolimus were prepared. The work concentrations of azoles, tacrolimus or everolimus were list in **Table 2**. The experimental, control, and negative groups were set up. Itraconazole, voriconazole, and posaconazole were added separately to the control group. In the negative control group, only spores and Dihydrorhodamine 123 were added to the test tube. The concentration of Dihydrorhodamine 123 was consistent with that of the experimental drugs. Itraconazole + tacrolimus, voriconazole + tacrolimus, posaconazole + tacrolimus, itraconazole + everolimus, voriconazole + everolimus, and posaconazole + everolimus were added to the experimental groups. The drugs were added in predetermined concentrations, which were the combined inhibitory concentration of drugs *in vitro*, to each group. About 5 µg/ml of Dihydrorhodamine 123 was added and the samples were placed on a shaker at 37°C at 130 rpm for 30 min. The spores were collected and cultured at 37°C at 130 rpm for 60 min and resuspended in 1 ml PBS. About 150 µl of the solution was subjected to flow cytometry (B53000, Beckman Coulter, USA) for several events. A total of 10000 events were performed and analyzed using CytExpert. The test was repeated thrice.

**Table 1:** MIC and FICIs results of tacrolimus and everolimus in combination with azoles.

Strain	MIC (µg/ml)					MIC [A/B(µg/ml)] (FICI)					
	TAC	EVL	ITR	VRC	POS	TAC/ITR	TAC/VRC	TAC/POS	EVL/ITR	EVL/VRC	EVL/POS
	*S. aurantiacum*										
CBS116910	8	2	4	0.25	0.5	4/1 (0.75)	4/0.125 (1)	4/0.25 (1)	1/2 (1)	0.5/0.25 (1.25)	1/0.25 (1)
	*S. apiospermum*										
L12E	4	4	2	1	0.5	**1/0.5 (0.5)**	**1/0.25 (0.5)**	**0.5/0.125 (0.375)**	**1/0.125 (0.375)**	**1/0.125 (0.375)**	**1/0.125 (0.5)**
CBS116899	4	1	4	1	8	**1/0.25 (0.3125)**	**1/0.25 (0.5)**	**0.5/1 (0.25)**	0.5/1 (0.75)	0.5/0.25 (0.75)	0.5/4 (1)
D13d	32	2	8	4	4	**2/1 (0.1875)**	**2/1 (0.3125)**	16/2 (1)	**0.5/1 (0.375)**	**0.5/0.5 (0.375)**	**0.5/1 (0.5)**
D13f	32	4	8	4	0.5	**4/2 (0.375)**	**0.5/1 (0.2656)**	4/0.25 (0.625)	**1/0.5 (0.3125)**	**1/0.125 (0.2813)**	1/0.25 (0.75)
D13g	16	2	8	0.5	0.5	8/4 (1)	8/0.125 (0.75)	8/0.25 (1)	**0.5/2 (0.5)**	**0.5/0.125 (0.5)**	1/0.125 (0.75)
SDB-l	32	4	8	1	2	16/4 (1)	8/0.5 (0.75)	2/1 (0.5625)	**1/2 (0.5)**	**1/0.25 (0.5)**	**1/0.25 (0.375)**
	*S. boydii*										
CBS101.22	8	2	2	1	0.5	**1/0.5 (0.375)**	**1/0.25 (0.375)**	**0.5/0.125 (0.3125)**	**0.5/0.5 (0.5)**	**0.25/0.125 (0.25)**	**0.5/0.125 (0.5)**
D13a	4	2	8	8	1	**1/2 (0.5)**	**1/1 (0.375)**	**1/0.25 (0.5)**	1/2 (0.75)	1/8 (1.5)	1/0.25 (0.75)
D13b	4	2	8	1	0.5	1/4 (0.75)	**1/0.25 (0.5)**	**0.5/0.125 (0.375)**	**0.5/2 (0.5)**	**0.5/0.25 (0.5)**	**0.5/0.125 (0.5)**
D13c	16	4	8	0.5	4	**4/2 (0.5)**	**2/0.125 (0.375)**	**2/0.25 (0.1875)**	2/2 (0.75)	2/0.125 (0.75)	**1/1 (0.5)**
M013	8	2	2	0.5	4	**1/0.5 (0.375)**	**1/0.125 (0.375)**	8/1 (1.25)	1/0.5 (0.75)	**0.5/0.125 (0.5)**	**0.5/1 (0.5)**
	*S. dehoogii*										
CBS117406	4	4	2	8	0.25	4/0.5 (1.25)	4/1 (1.125)	0.5/0.25 (1.125)	**0.5/0.25 (0.25)**	2/8 (1.5)	0.5/0.25 (1.125)
	*L. prolificans*										
D13h	8	4	8	16	8	4/2 (0.75)	**1/2 (0.25)**	**1/2 (0.5)**	2/2 (0.75)	2/8 (1)	2/2 (0.75)
	*S. minutisporum*										
SDB-i	4	2	8	0.5	1	**0.5/1 (0.25)**	**0.5/0.125 (0.375)**	2/0.5 (1)	1/2 (0.75)	0.5/0.25 (0.75)	1/0.25 (0.75)

Synergy (FICI of ≤ 0.5); no interaction (indifference, 0.5 < FICI ≤ 4). TAC, tacrolimus; EVL, everolimus; ITR, itraconazole; VRC, voriconazole; POS, posaconzaole. The combinations MICs that showed synergy (FICI ≤ 0.5) are shown in bold.

**Table 2:** ROS activity and apoptosis experiments with different drug concentrations.

Control groups	Concentration(µg/ml)	Experimental groups	Concentration(µg/ml)
ITR	2	ITR/TAC	0.25/0.5
VOR	0.5	VOR/TAC	0.125/0.5
POS	0.25	POS/TAC	0.06/0.25
TAC	2	ITR/EVL	0.25/0.5
EVL	2	VOR/EVL	0.06/0.5
		POS/EVL	0.06/0.5

TAC, tacrolimus; EVL, everolimus; ITR, itraconazole; VOR, voriconazole; POS, posaconazole.

### Combined Drug Effect on Efflux Pump

*S. apiospermum* (L12E) was cultured in SDB medium at 37°C at 130 rpm overnight and finally resuspended in PBS solution to prepare a final solution concentration of 1 × 10^8^/ml. The solution was kept on a shaker for 2 hours so that the energy is completely consumed. Negative control and experimental groups were set up. The negative control group included two subgroups - no glucose and antifungal drugs subgroup and glucose but no antifungal drugs subgroup. The glucose concentration (5%) in the two subgroups was the same. The experimental group contained both glucose and antifungal drugs (concentrations were referred in **Table 2**). First, the fluorescent agent Rhodamine 6G (10 mM) was added to all test tubes, the tubes were shaken at 37°C at 200 rpm for 50 min, and, then, kept in an ice bath for 10 min. The tubes were, then, centrifuged at 3000 rpm for 5 minutes. Then, the solution was washed with PBS three times to resuspend the spores, and a predetermined concentration of drug and glucose solution was added to each test tube. The solutions were, then, immediately transferred to a 96-well plate. An enzyme labeling instrument (DNM-9602), purchased from Perlong, Beijing, was used to detect the absorbance (488 nm) every ten minutes for the first 50 min.

### The Effect of Combined Drugs on Apoptosis

The *S. apiospermum* (L12E) was cultured at 37°C in SDA for three days. The spores of L12E were accurately counted and diluted in 10 ml SDB to make a final concentration of 5 × 10^6^/ml. The negative control, azoles control, and the combined experimental groups of azoles, tacrolimus, and everolimus were set. In the negative control group, Annexin-V-FITC and PI (Propidium Iodide) were added to the test tube. In the azole control group, itraconazole, voriconazole, and posaconazole were added to three different test tubes. Two control groups, tacrolimus and everolimus, were also set up in the experiment. The drug concentrations were the same as those used in the above experiments (**Table 2**). After adding a predetermined concentration of the drug, the solution was shaken for 1 h at 37°C at 150 rpm. The spores were collected by washing with PBS. About 5 µl Annexin-V-FITC and 10 µl PI fluorescent agents were added and let to react in the dark for 15 min. The solution was, then, transferred to flow cytometry tubes and 10000 events were collected. The test was repeated three times.

### *In Vivo* Efficacy of Tacrolimus or Everolimus Alone and in Combination With Azoles

Since *S. apiospermum* (L12E) was isolated from a hyalohyphomycosis patient who was successfully cured by voriconazole and tacrolimus, we also used it to establish the infected model. To evaluate the synergistic effect of tacrolimus or everolimus combined with azoles *in vivo*, *G. mellonella* (sixth instar larvae) was infected with *S. apiospermum* (L12E), and then the infected *G. mellonella* were treated with the experimental drug combinations of tacrolimus, everolimus, and azole drugs. A total of 12 experimental groups were established, with 20 *G. mellonella* (∼300 mg, Sichuan, China) in each group. Before the experiment, they were stored in a container at 37°C and cultured in the dark. The spores of L12E were cultured on SDA at 37°C for 3 days and dissolved in sterile water to 1 × 10^7^ spores/ml. To evaluate the *in vivo* effects of tacrolimus and everolimus alone and in combination with azoles, the following experimental groups were included: tacrolimus group, everolimus group, itraconazole group, voriconazole group, posaconazole group, tacrolimus + itraconazole group, tacrolimus + voriconazole group, tacrolimus + posaconazole group, everolimus + itraconazole group, everolimus + voriconazole group, and everolimus + posaconazole group. The concentration of drugs was the combined inhibitory concentration of drugs *in vitro*. The control group included the untreated group, sterile normal saline group, and conidia suspension group. The sterile normal saline group and conidia suspension group were injected with 10 μl of corresponding solution. No processing was done to the untreated group. The injection site was disinfected with a cotton swab dipped in alcohol, and the conidia suspension and therapeutic and control solutions were injected into the larva through the last gastropod on the left side of the larva by using the Hamilton syringe (25 gauge, 50 μl). The larvae were cultured in a plastic box at 37°C in the dark. The survival of the larvae was observed and recorded at the same time every day for 6 days.

### Statistical Analysis

All test results were analyzed using CytExpert and GraphPad Prism 8 software, and the data are presented as mean ± SEM. In the ROS activity experiment, the Shapiro-Wilks test was used to confirm the normal distribution of the data (*P* > 0.5), except for the experimental group of everolimus + itraconazole. The differences between groups were analyzed using the T-test, and those in the everolimus and itraconazole groups were analyzed using the nonparametric T-test. In the experiment of exploring the effect of drugs combination on spore apoptosis at different stages, the Shapiro-Wilks test was performed to confirm that all data were normally distributed (*P* > 0.5). The differences between groups were analyzed using the T-test.

## Results

### Drug Sensitivity *In Vitro*

**Figure 1:**
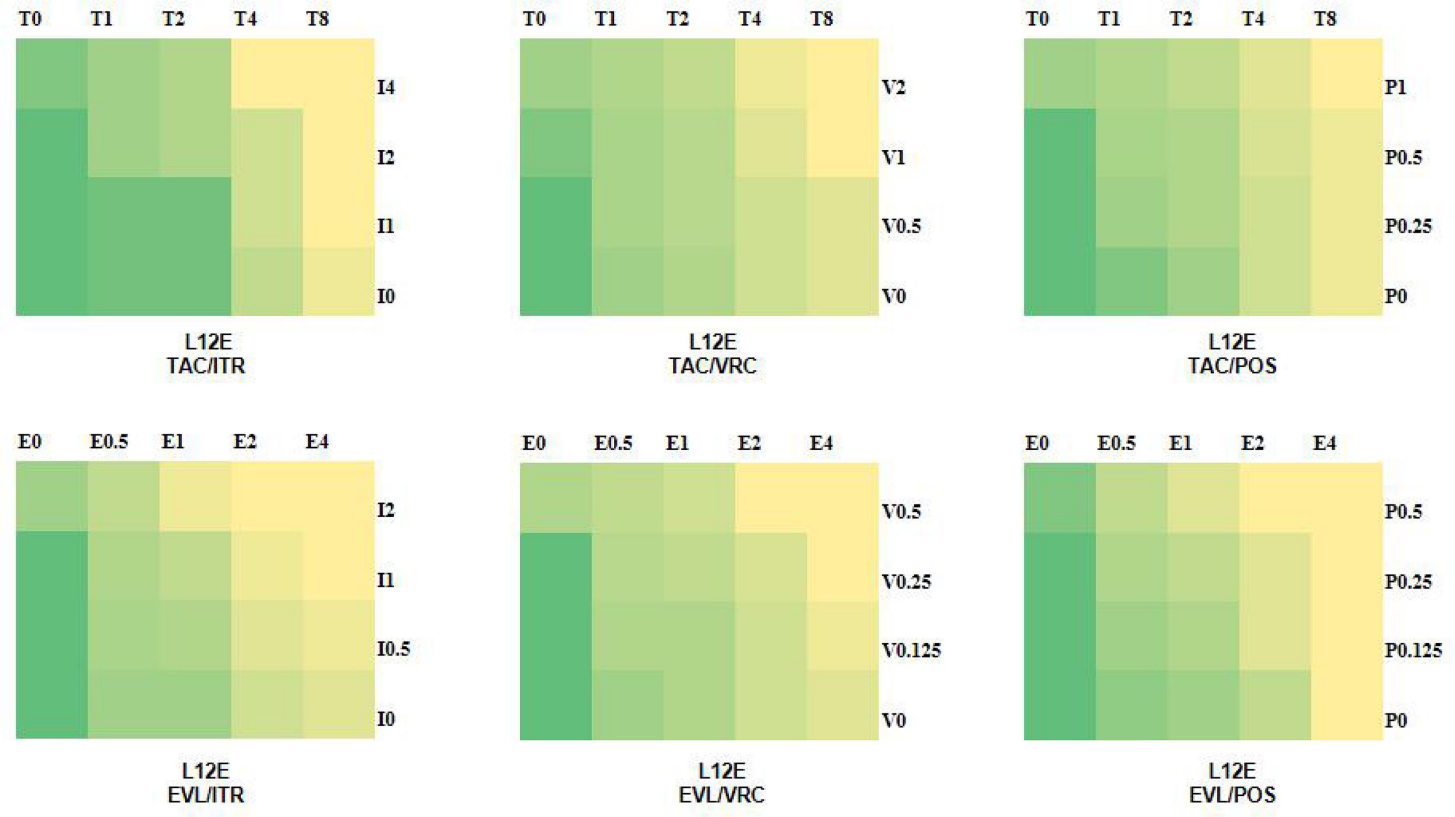
Schematic diagram of drug sensitivity. The colors of green to yellow represent the amount of fungal growth in the various drug combination concentrations. Green indicates good fungal growth, yellow indicates poor fungal growth. T, tacrolimus; E, everolimus; I, itraconazole; V, voriconazole; P, posaconazole; and the number after the letter represents the concentration (µg/ml).

The MIC ranges of tacrolimus, everolimus, posaconazole, itraconazole, and voriconazole were 4-32 µg/ml, 1-4 µg/ml, 0.25-8 µg/ml, 2-8 µg/ml, and 0.25-8 µg/ml, respectively. For *Scedosporium* spp., the resistant break points of itraconazole, voriconazole, and posaconazole were 8, 4, and 2, respectively (Carvalhaes et al., 2021). It was observed that the tested *Scedosporium* and *Lomentospora* were resistant to the three azoles (posaconazole, itraconazole, and voriconazole). The *in vitro* results showed that the combinations of tacrolimus with itraconazole, voriconazole, and posaconazole showed synergistic effects on 9 strains (60%), 10 strains (73%), and 7 strains (47%), respectively, and the combination of everolimus with itraconazole, voriconazole, and posaconazole showed synergistic effects on 8 strains (53%), 8 strains (53%), and 7 strains (47%), respectively (**Table 1**). There was no antagonism in the combination groups. According to the *in vitro* drug sensitivity test and checkerboard method, the sensitivity diagrams of strain L12E are shown in **Figure 1**.

### ROS Activity

**Figure 2:**
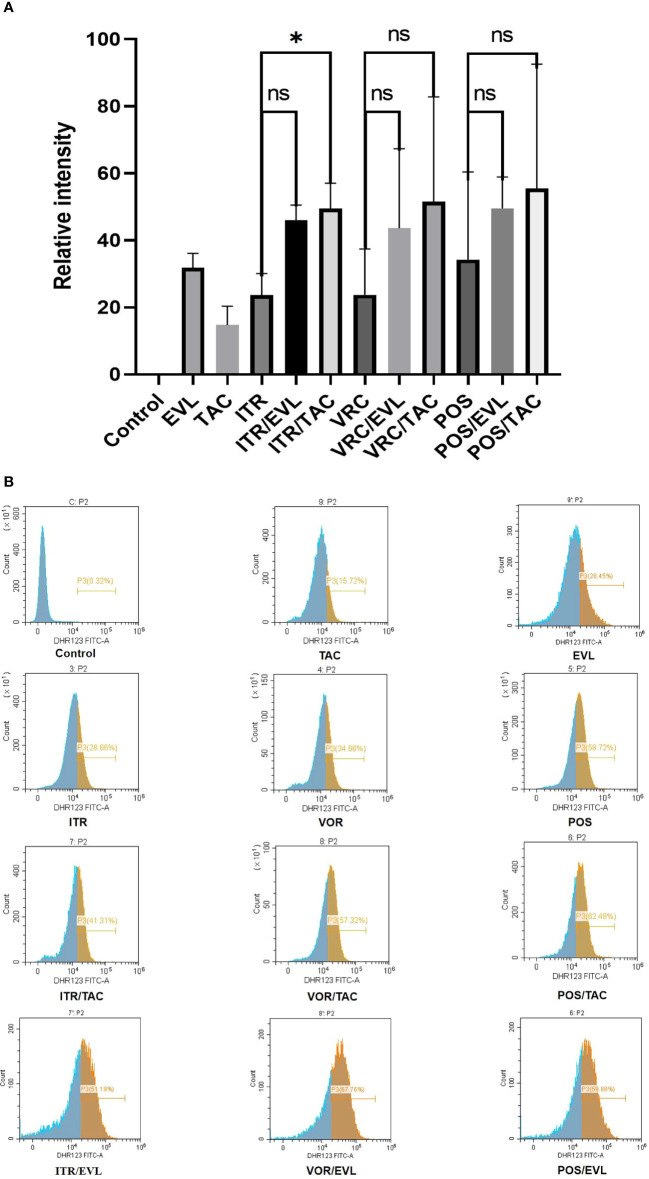
Changes in ROS generation ratio across the single and combined drug groups. **(A)** The proportion of Dihydrorhodamine 123 oxidized by ROS in the control and experimental groups and related statistical analysis. **(B)** In flow cytometry analysis, the abscissa represents the relative fluorescence intensity and the ordinate represents the spore count. The brown area represents the percentage of spores (P3) with emitted fluorescence after Dihydrorhodamine 123 was oxidized to Rhodamine 123. The peak value in the brown area indicates the largest number of oxidized spores under the corresponding fluorescence intensity. TAC, tacrolimus; EVL, everolimus; ITR, itraconazole; VRC, voriconazole; POS, posaconazole. *P < 0.05; ns, no significance.

The ROS activity level was higher in the combination of azoles with tacrolimus or everolimus compared with single drug. The ROS activity levels of itraconazole, voriconazole and posaconazole were 23.66%, 23.78% and 34.28% respectively. When tacrolimus and everolimus were used alone, the ROS activity levels were 14.76% and 31.84% respectively. When itraconazole, voriconazole and posaconazole were combined with tacrolimus, compared with azoles alone, ROS activity levels increased by 25.82%, 27.82% and 21.23% respectively. When itraconazole, voriconazole and posaconazole were combined with everolimus, compared with azoles alone, ROS activity levels increased by 22.40%, 19.86% and 15.30% respectively. There was a significant difference between the itraconazole and tacrolimus combined experimental groups (*, *P* < 0.05) (**Figure 2**).

### Effect on Drug Efflux

**Figure 3:**
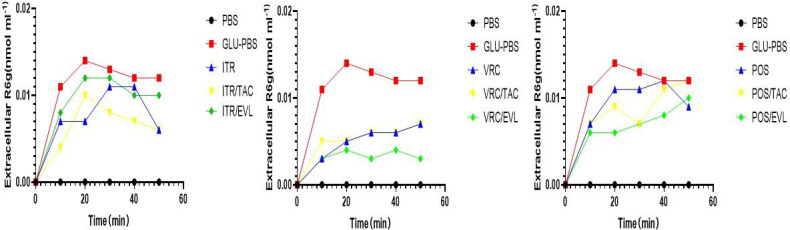
The evaluation of efflux pump function. The effect of TAC combined with azoles on the Rhodamine 6G outflow of *S. apiospermum* (L12E). The extracellular Rhodamine 6G concentration of *S. apiospermum* (L12E) was determined using spectrophotometry. Energy-dependent Rhodamine 6G efflux was quantified by adding glucose, azole, and TAC with Rhodamine 6G (10 mM) or Rhodamine 6G (10 mM) in glucose-free PBS and measuring the absorbance of the supernatant at 488 nm. TAC, tacrolimus; EVL, everolimus; ITR, itraconazole; VRC, voriconazole; POS, posaconazole. The values represent the mean and SD of three independent experiments.

We used spectrophotometry to determine the extracellular Rhodamine 6G concentration of L12E in the absence or presence of tacrolimus and everolimus after adding the three azoles (itraconazole, voriconazole, and posaconazole). After glucose was added to the conidial suspension, the extracellular Rhodamine 6G concentration increased sharply. All tests were repeated three times and the average was used as the final result. When L12E was maintained in PBS without glucose, there was no Rhodamine 6G secretion outside the cells. Compared to the azole-only groups, when tacrolimus or everolimus were combined with itraconazole, voriconazole, and posaconazole, the results showed no significant differences, suggesting that the synergistic effects of tacrolimus and everolimus with azoles have no significant effect on the efflux pump (**Figure 3**).

### Apoptosis in Different Periods

**Figure 4:**
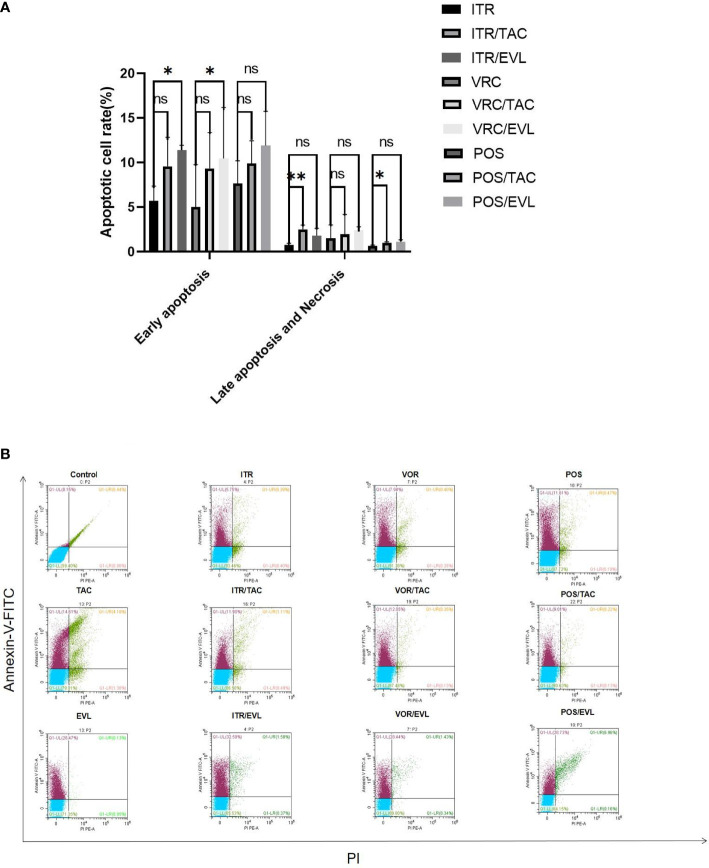
The effect of drug treatment on apoptosis. **(A)** The proportion of spores undergoing apoptosis at different stages of apoptosis in the control group and the experimental group and related statistical analysis. **(B)** In flow cytometry analysis, Annexin-V-FITC and PI fluorescents emit different wavelengths of fluorescence in the early and late stages of apoptosis. The lower left quadrant represents living cells; the upper left quadrant represents early apoptotic cells; the upper right quadrant represents necrotic and late apoptotic cells. TAC, tacrolimus; EVL, everolimus; ITR, itraconazole; VRC, voriconazole; POS, posaconazole. *P < 0.05; **P < 0.01; ns, no significance.

Annexin-V can bind to phosphatidylserine (PS) with high affinity and to the cell membrane of early apoptotic cells through the phosphatidylserine exposed outside the cells. PI is a nucleic acid dye. It cannot penetrate the complete cell membrane of normal cells or early apoptotic cells but can penetrate the cell membrane of late apoptotic and necrotic cells and stain the nucleus red. Therefore, when Annexin-V was combined with PI, PI was excluded from the living and early apoptotic cells, while late apoptotic and necrotic cells were stained by FITC and PI (Zhang and Liang, 2014). When azoles were used in combination with tacrolimus or everolimus, the levels of early apoptotic, necrotic, and late apoptotic cells increased significantly. There were significant differences in late apoptosis cells after itraconazole (**, *P* < 0.01) and posaconazole (*, *P* < 0.05) were combined with tacrolimus separately. There was a significant difference in early apoptosis when itraconazole (*, *P* < 0.05) and voriconazole (*, *P* < 0.05) were combined with everolimus separately (**Figure 4**).

### The Effects of the Drug Combinations on Infected *G. mellonella In Vivo*

**Figure 5:**
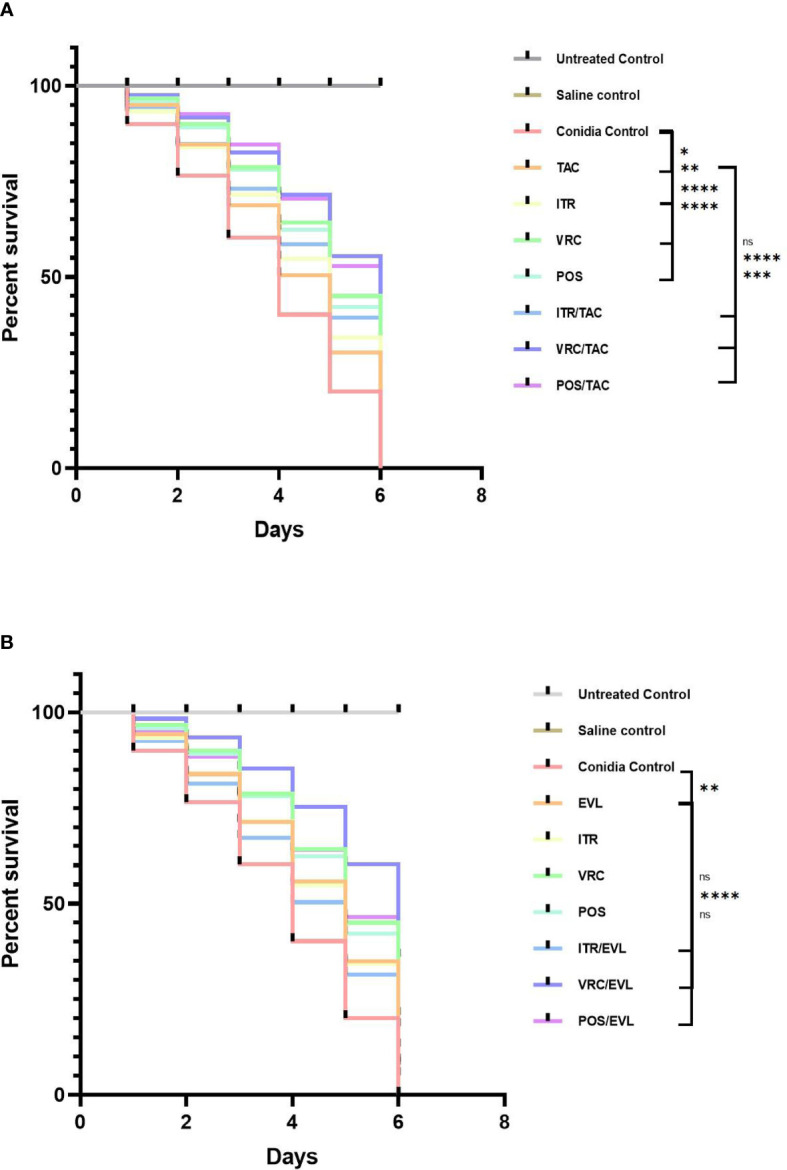
The survival curves of larvae infected with *Scedosporium* after different interventions. **(A)** TAC group **(B)** EVL group. *G. mellonella* infected with *Scedosporium*. Tacrolimus, everolimus, and azoles alone or in combination with tacrolimus or everolimus can significantly improve the survival rate of larvae. Tacrolimus and everolimus combined with azoles showed a synergistic effect on *Scedosporium* infection. TAC, tacrolimus; EVL, everolimus; ITR, itraconazole; VRC, voriconazole; POS, posaconazole. *P < 0.05; **P < 0.01; ***P = 0.0001; ****P < 0.0001; ns, no significance.

For the *S. apiospermum* (L12E)-infected groups, we calculated the survival rates of the control and experimental groups after 5 days. In the control group, the survival rates of larvae treated with tacrolimus, everolimus, itraconazole, voriconazole, and posaconazole were 16.7%, 23.3%, 15%, 38.3%, and 31.7%, respectively. In the experimental group of tacrolimus combined with azoles, the survival rates of larvae treated with tacrolimus + itraconazole, tacrolimus + voriconazole, and tacrolimus + posaconazole were 30%, 53.3%, and 48.3%, respectively. In the experimental group of everolimus combined with azoles, the survival rates of larvae treated with everolimus + itraconazole, everolimus + voriconazole, and everolimus + posaconazole were 25%, 60%, and 45%, respectively. The survival rate of larvae was significantly improved with tacrolimus alone, everolimus alone, azoles alone, and tacrolimus and everolimus combined with azoles (*, *P* < 0.05 for the tacrolimus group; **, *P* < 0.01 for the everolimus group and itraconazole group; ***, *P* = 0.0001 for the tacrolimus + posaconazole group; ****, *P* < 0.0001 for other groups except everolimus + itraconazole group, everolimus + posaconazole group, and tacrolimus + itraconazole group; **Figure 5**).

## Discussion

*Scedosporium* and *Lomentospora* species are widely distributed in polluted environments, such as swamps, wetlands, sewage, saltwater, rot, and so on. *Scedosporium*/*Lomentospora* infection is reported in several countries, including Europe, South America, and Australia. In Spain and Australia, *S. apio*spermum is the second most common filamentous fungus causing clinical infection after *Aspergillus* (Alastruey-Izquierdo et al., 2013; Slavin et al., 2015).

Because of the resistance of *Scedosporium* and *Lomentospora* species to a variety of antifungal drugs, it is very difficult to treat infections with *Scedosporium* and *Lomentospora* species. *S. apiospermum* showed low or even no sensitivity to a variety of antifungal drugs *in vitro*, such as amphotericin B, nystatin, itraconazole, flucytosine, fluconazole, terbinafine, and ketoconazole (Li et al., 2017). Voriconazole is the first-line treatment for hyalohyphomycosis caused by *Scedosporium* species (He et al., 2015). The combination of tacrolimus and voriconazole can effectively inhibit the reproduction of fungi, such as *Aspergillus fumigatus* and *Cryptococcus*, and control the severity of infection (Cheng et al., 2020). At present, there is no report on the application of the combination of everolimus and different azoles. However, everolimus is often used to avoid rejection after renal transplantation (Pascual et al., 2018). Infection with *Scedosporium* after renal transplantation followed by immunosuppressants is very common all over the world (Uno et al., 2014; Torres-Sanchez et al., 2018). Therefore, exploring the synergistic effects of tacrolimus or everolimus combined with azole and their potential underlying mechanism of action may enable clinical trials for the combination treatment to combat infection caused by *Scedosporium* and *Lomentospora* species.

In this study, we chose 15 clinical strains of *Scedosporium* and *Lomentospora* species for the *in vitro* study. The results showed that tacrolimus or everolimus combined with azoles had synergistic effects. The results of the *in vitro* experiments showed that the MIC of azole drugs decreased significantly. When tacrolimus was combined with the three azoles, the MIC range decreased from 2-8 µg/ml to 0.25-4 µg/ml for itraconazole. For voriconazole and posaconazole, the MIC range decreased from 0.25-8 µg/ml to 0.125-2 µg/ml. The MIC range of tacrolimus decreased from 4-32 µg/ml to 0.5-16 µg/ml. When everolimus was combined with the three azoles, the MIC range decreased from 2-8 µg/ml to 0.125-2 µg/ml for itraconazole, 0.25-8 µg/ml to 0.25-8 µg/ml for voriconazole, and 0.125-8 µg/ml to 0.125-4 µg/ml for posaconazole, respectively. The MIC range of everolimus decreased from 1-4 µg/ml to 0.25-2 µg/ml. The decreased MIC values indicate lower drug dosages might effectively combat the pathogen without weaken therapeutic effect. *In vivo*, using the *G*. *mellonella* model, we also observed the synergistic effect of azoles and tacrolimus or everolimus. Therefore, when patients using tacrolimus for immunosuppressant or everolimus for cancer treatment are infected with *Scedosporium* or *Lomentospora* species, we might choose azoles for the synergistic effect to treat this type of infection.

Previous studies have shown that cyclosporin and tacrolimus inhibit calcineurin, which has toxic effects on *Candida albicans* and *Cryptococcus neoformans* (Cruz et al., 2001). Tacrolimus combined with itraconazole or ketoconazole has a synergistic effect on *Malassezia* (Sugita et al., 2005), and the combination of tacrolimus and voriconazole shows synergistic inhibitory activity against *Aspergillus* biofilms (Gao and Sun, 2015). To further explore the mechanisms of action underlying the combined action of azoles and tacrolimus on *S. apiospermum*, we examined their influence on the efflux pump, apoptosis, and ROS activity levels. Our Rhodamine 6G data showed no significant changes between itraconazole, voriconazole, and posaconazole alone or in combination with tacrolimus and everolimus, suggesting that tacrolimus and everolimus combined with azoles have no significant effect on the efflux pump. However, when azoles were combined with tacrolimus and everolimus against *S. apiospermum*, the levels of ROS activity and apoptosis increased, especially in the itraconazole and tacrolimus group, which showed that the levels of ROS activity (*P* < 0.05) and late apoptosis (*P* < 0.01) were significantly higher than those in the itraconazole group. The everolimus + itraconazole and everolimus + voriconazole groups showed a significant increase in early apoptosis compared to itraconazole and voriconazole alone (*P* < 0.05). Also, the tacrolimus + posaconazole group showed a significant increase in late apoptosis compared to posaconazole alone (*P* < 0.05). The results showed that tacrolimus or everolimus combined with azoles (itraconazole, voriconazole, and posaconazole) could increase the level of ROS activity and apoptosis. The potential mechanism might be due to the inhibition of TOR signaling pathway or cacineurin pathway which governs important processes in fungal physiology (Bekeschus et al., 2019; Li et al). And from our *in vivo* and *in vitro* results, everolimus alone exhibits a potential anti- *Scedosporium*/*Lomentospora* effect which may be associated with the role of everolimus in decreasing the activity of the TOR pathway. However, the mechanism of action of synergy and inhibition of everolimus needs more research in future.

In conclusion, from our *in vitro* and *in vivo* study, tacrolimus and everolimus combined with azoles may have a synergistic effect against *Scedosporium/Lomentospora*, improving the drug activity of azoles. And everolimus alone also inhibited the growth of *Scedosporium/Lomentospora* as shown using the *in vitro* drug sensitivity test. The synergistic mechanisms of action of tacrolimus and everolimus combined with azoles might trigger the production of excessive ROS activity and apoptosis. Through *in vivo* experiments, we also confirmed that the combination of tacrolimus or everolimus and azoles had a synergistic effect on *G. mellonella.* As shown from the current research, the synergistic effect of tacrolimus or everolimus with azoles might help the patients with a better prognosis. However, further research with a larger sample size and the elucidation of the mechanism of action is needed for better understanding the treatment value.

## Data Availability Statement

The original contributions presented in the study are included in the article/supplementary material. Further inquiries can be directed to the corresponding author.

## Ethics Statement

Written informed consent was obtained from the individual(s) for the publication of any potentially identifiable images or data included in this article.

## Author Contributions

ZW performed all *in vitro* experiments. ML performed the *in vivo* study. ZW and ML wrote the manuscript, and YS provided general guidance and revised the manuscript. LLiu provided some test methods, LLi helped with flow cytometry, and LT helped cultivate the strains. All authors contributed to the article and approved the submitted version.

## Funding

This work was supported by Grant No. WJ2021M261 (Yi Sun) from the Health Commission of the Hubei Province scientific research project and Grant No. 2019CFB567 (YS) from the Natural Science Foundation of the Hubei Province. The funder had no role in study design, data analysis, the decision to publish, or preparation of the manuscript.

## Conflict of Interest

The authors declare that the research was conducted in the absence of any commercial or financial relationships that could be construed as a potential conflict of interest.

## Publisher’s Note

All claims expressed in this article are solely those of the authors and do not necessarily represent those of their affiliated organizations, or those of the publisher, the editors and the reviewers. Any product that may be evaluated in this article, or claim that may be made by its manufacturer, is not guaranteed or endorsed by the publisher.
